# Boost Me: Prevalence and Reasons for the Use of Stimulant Containing Pre Workout Supplements Among Fitness Studio Visitors in Mainz (Germany)

**DOI:** 10.3389/fpsyg.2018.01134

**Published:** 2018-07-17

**Authors:** Matthias Dreher, Tobias Ehlert, Perikles Simon, Elmo W. I. Neuberger

**Affiliations:** Department of Sports Medicine, Rehabilitation and Disease Prevention, Johannes Gutenberg University Mainz, Mainz, Germany

**Keywords:** pre workout booster, stimulants, fitness studios, nutritional supplements, prevalence

## Abstract

The rapidly increasing interest in fitness related sports over the past few years has been accompanied by a booming industry of nutritional supplements. Many of these substances have unproven benefits and are even potentially harmful to the user. The aim of this study was to determine the prevalence and reasons for nutritional supplement (NS) use among fitness studio visitors in Mainz (Germany), emphasizing new multi-ingredient based supplements such as pre workout boosters (PWBs). Some of the PWBs contain stimulants such as DMAA, N,α-DEPEA, DMAE and DMBA with so far unknown risks, harms and benefits. Four-Hundred and Ninety Two participants in 13 fitness studios completed a questionnaire on the use of nutritional supplements. Descriptive statistics and chi-square tests were used to examine differences in supplement use regarding training- and intake-reasons. About 57.0% of the participants reported the use of NS during the last 4 weeks. The all-time prevalence of creatine use was 28.7%, whereas 12.2% of the participants stated creatine use during the past 4 weeks. The all-time prevalence of PWB intake was 25.8%, whereas the last month prevalence was 11.8%. Among the group of PWB users, 20.5% stated to search specifically for substances such as DMAA, N,α-DEPEA, DMAE or DMBA. Logistic regression analysis showed positive relations between creatine use and the predictor variables gender, strength training and bodybuilding, as well as the stated exercisers' training reasons to increase physical- and sports-performance, and quality of life. PWB consumption was related to the variables gender, training frequency, and the reason for sports performance enhancement. Specific ingredient focus was related to the predictor variables competition participation and increase of mental performance. The results of the study show a high prevalence of PWB consumption among fitness studios visitors, which is comparable with creatine use. The predicting variables for consumption seem to be slightly different between the supplements, especially if the users are searching for stimulating agents. The current findings help to create preliminary consumption patterns and can help to identify potential endangered fitness studio visitors for prevention and risk communication, especially for PWBs.

## Introduction

The nutritional supplement (NS) industry is a rapidly growing market. From 1994 to 2012, the number of nutritional supplements available in the USA increased from an estimated 4,000 to more than 55,000 (Cohen, 2012a). Globally, the market volume for NS was about $132.8 billion in 2016 and it is expected to reach $220.3 billion by 2022 (Zion Market Research, 2017). According to the Dietary Supplement Health and Education Act (DSHEA) of 1994, NS are products to supplement the normal diet, including vitamins, minerals, probiotics, botanical extracts, and other ingredients intended for ingestion as a pill, capsule, tablet, or in liquid form (National Institute of Health, 2017). Despite the evidence that even physically active people have no physiological need for NS if they have a balanced diet, the use of NS greatly increased in the past few years (Thomas et al., 2016) and the popularity for the use of NS is high among the general population, in elite athletes, as well as recreational athletes. As reviewed by Solheim et al. (2017), the NS use in athletes ranges from 48 to 100% depending on stage of life, gender, age, sports type and discipline, time of season, and geography. In the U.S. population, about 43% of men and 54% of women used NS in the past month (Bailey et al., 2011). In a German cohort of 25,544 men and women, aged 35–65, the regular use of NS was ~41% in men and ~47% in women (Reinert et al., 2007). Lieberman et al. (2015) studied NS use among U.S. college students and reported that about 66% of the study population used one or more NS at least once a week, over 6 months prior to the survey. In a Danish study of elite athletes who train in fitness studios, 92.6% of females and 85.0% of males reported the use of NS within the preceding 7 days (Solheim et al., 2017). Among common fitness studio visitors, the regular use of supplements ranges between 30 and 100% (Morrison et al., 2004; Oliver et al., 2008; Goston and Correia, 2010; Bianco et al., 2011; El Khoury and Antoine-Jonville, 2012; Saeedi et al., 2013; Solheim et al., 2017). Focused on age, younger fitness studio visitors showed a higher NS intake than adults (Morrison et al., 2004; Goston and Correia, 2010; El Khoury and Antoine-Jonville, 2012; Saeedi et al., 2013). Furthermore, men showed higher prevalence than females, if focused on supplements which suggest a performance enhancing effect (El Khoury and Antoine-Jonville, 2012; Saeedi et al., 2013; Solheim et al., 2017). Krumbach et al. (1999) and Lieberman et al. (2015) showed for college students and Scofield and Unruh (2006) for adolescents, that the aspect of increasing performance constitutes an important reason for NS intake. For older adults, disease protection and prevention are the main reasons for NS intake identified by Morrison et al. (2004) and Saeedi et al. (2013). Further NS intake reasons are to shorten the recovery time under a mental perspective, as well as the increase of mental and cognitive performance, e.g., with caffeine, which is described among young elite athletes and university sports students (Sato et al., 2012; Dietz et al., 2013). In fitness sports training specific parameters are related to NS intake. Goston and Correia (2010) and Oliver et al. (2008) showed that higher training frequency and longer training duration are accompanied with higher NS intake, whereas El Khoury and Antoine-Jonville (2012) stated a peak for NS intake for fitness studio visitors which have a training experience over 1 year, train between 3 and 5 times a week, and train between 1 and 2 h per session. In summary, the main reasons for athletes to use NS seem to be improvements in overall health status, to compensate for an assumedly poor diet, to enhance cognitive or physical performance, to increase energy capacity, and to reduce weight (Maughan et al., 2011; Knapik et al., 2016b). Physical performance, as well as the recovery from sporting activities, depends on an appropriate diet and timing of food and fluid intake (Thomas et al., 2016). A small number of legal nutritional substances have shown beneficial effects on sports performance, including creatine, caffeine, sodium bicarbonate, beta-alanine, and, at least in non-elite athletes, nitrate supplementation (Thomas et al., 2016). Especially creatine is a widely used and well-studied performance-enhancing supplement (Knapik et al., 2016a). However, the use of NS can possibly have negative consequences as the long-term safety profile of NS supplementation is not available for most substances, especially not for mixed consumption of NS (Maughan et al., 2004). According to a meta-analysis by Bjelakovic et al. (2012), the use of antioxidant supplements beta-carotene and vitamin E increases mortality. Moreover, NS can be contaminated with non-declared substances (Geyer et al., 2004) or a NS manufacturer could willingly include physiologically active substances with unknown safety profiles (Cohen, 2014). Unlike prescription medications, nutritional supplements can be marketed without proven evidence of efficacy or safety, and therefore without premarket approval (Cohen, 2012a).

One of the current NS product types with a relevant market share are so called “pre workout boosters” (PWBs) which are sub classified by manufacturers e.g., in “hardcore boosters.” These supplements are being used to increase energy, extend endurance, and boost muscle gains (Eudy et al., 2013). The generally water soluble powders are consumed about 30 min before training and typically consist of a large number of diverse substances including creatine, caffeine, beta alanine, arginine, taurine, and phosphates (Eudy et al., 2013). Additionally, PWB often contain ingredients with allegedly botanical sources, such as 1,3-dimethylamylamine (DMAA) or β-methylphenylethylamine (BMPEA), whereas the only known source is chemical manufacturing (Austin et al., 2014; Cohen et al., 2016). DMAA has effects similar to ephedrine and amphetamine (Austin et al., 2014), including an increase in arterial blood pressure, vasoconstriction, tachycardia and bronchodilation (Venhuis and de Kaste, 2012). As reviewed by Cohen (2012b), DMAA was introduced in 1948 as a nasal inhaler for rhinitis, but was withdrawn from the US market over 40 years ago. In 2006, the substance was re-introduced into the market as a component of dietary supplements. As an ingredient in energy boosters and weight loss supplements, DMAA was sold in more than 200 products with over $100 million sales in 2010 (Cohen, 2012b). Numerous case reports indicate that a high dose ingestion of DMAA could have serious adverse effects, including death (Eliason et al., 2012; Gee et al., 2012; Young et al., 2012; Archer et al., 2015). Since 2011 and 2012, DMAA was banned in several countries such as Canada, New Zealand, United States, and the European Union. Following the ban, novel synthetic stimulants were added to the supplements. These included N,α-diethyl-phenylethylamine (N,α-DEPEA) (Cohen, 2014), 1,3-dimethylbutylamine (DMBA) (Cohen et al., 2015), and β-methylphenylethylamine (BMPEA) (Cholbinski et al., 2014; Cohen et al., 2016). The mechanism of action of newly designed substances is usually unknown and no safety studies were conducted (Cohen et al., 2016).

According to a systematic review by Dunn (2017), a total of 16 studies evaluated acute and long-term physiological effects of DMAA, investigating 201 healthy participants, including 126 males, 35 females, and 40 control subjects. Whitehead et al. (2012) and Bloomer et al. (2013) studied the effect of DMAA in combination with caffeine for 10 or 12 weeks, respectively, on blood pressure and further bloodborne markers in a double-blinded, randomized, placebo-controlled study design. After the intervention, no physiologically relevant differences were detected in the treatment groups compared to the control groups. But the use of supplements containing DMAA and caffeine increases blood pressure in the short-term with a peak at 60–90 min post ingestion with minimal effects on heart rate (Bloomer et al., 2011; Farney et al., 2012; McCarthy et al., 2012a,b). The daily ingestion of DMAA containing supplements for periods between 14 days and 12 weeks had no pathological consequences in the studied populations (Bloomer et al., 2011, 2013; Farney et al., 2012; McCarthy et al., 2012a,b).

It is mentionable that participants were not allowed to ingest additional caffeine containing beverages during the study period. The mixed consumption of different stimulant-like substances, including caffeine in high dosages can be highly problematic and can lead to severe adverse effects including cardiovascular risks (Cohen, 2012b; Schilling et al., 2013). The composition and concentration of the ingredients of PWBs is often not declared in detail and the amount of active substances can vary widely (Cohen et al., 2016). Users are often unaware of the possible negative consequences of NS use (Maughan et al., 2004, 2011) and unthoughtful consumption can have adverse effects. Geller et al. (2015) analyzed supplement-related emergency room visits in the general population. Based on representative surveillance data of 3,667 cases from 2004 to 2013, the author's calculation estimates an average of about 23,000 hospital visits related to adverse effects of nutritional supplements in the United States. After exclusion of children that ingested nutritional supplements without supervision, weight loss and energy products were responsible for emergency room visits, and related to ~72% of the adverse events in the age group of 20–34. The adverse effects involved palpitations, chest pain or tachycardia.

To our knowledge, the use of pre workout boosters containing stimulants in the group of commercial fitness studio visitors has not been investigated yet. In this study, we assessed the general use of NS among fitness studio visitors in Mainz (Germany) considering the supplements, sociodemographic and anthropometric characteristics, frequency and reasons for NS use and fitness training. Our main focus was to obtain a widespread first impression of the prevalence for PWBs that contain not only common stimulants like caffeine or taurine but also synthetic stimulants such as DMAA, N,α-DEPEA, DMAE, or DMBA. Furthermore, we investigated if the intake is predictable by the surveyed variables. We compared PWB supplements with creatine which is a well-studied supplement, used for performance enhancement. The creation of a consumption pattern can help to identify potential endangered fitness studio visitors, and might be used for prevention and risk communication, especially for PWB use.

## Materials and methods

### Sample

A total of 492 fitness studio visitors took part in the study (Table 1 summarizes the basic characteristics). About 60% of the participants were male (*N* = 295). The majority of all surveyed athletes were in the age groups between 21 and 25 (37.0%), 15 and 20 (19.3%), and 26 and 30 (17.5%). Over 60% of the study population had A-levels or a higher educational degree. The BMI of the participants was calculated from self-reported height and weight. 305 athletes (62.0%) were defined as normal weight (18.5–24.9), followed by 131 (26.6%) participants classified as overweight (BMI 25.0–29.9). A BMI >30.0 was classified as obese, which pertains to 29 persons (5.9%). The average BMI of female athletes was 22.3 kg/m^2^ (SD ± 3.7), and of males 25.1 kg/m^2^ (SD ± 3.1). Importantly, in this population, the BMI does not necessarily describe the body composition regarding fat mass and fat free mass.

**Table 1:** Basic characteristics of 492 fitness studio visitors.

**Variable**	***N***	**(%)**
**GENDER**		
Male	295	59.96
Female	194	39.43
**AGE GROUPS**		
15–20	95	19.31
21–25	182	36.99
26–30	86	17.48
31–35	37	7.52
36–40	17	3.46
41–45	16	3.25
46–50	18	3.66
≥ 51	37	7.52
**SCHOOL EDUCATION**		
A-levels	304	61.79
**JOB TYPE**		
Student/pupil/apprentice	232	47.20
**FAMILY STATUS**		
Unmarried	396	80.49
**BMI**		
< 18.5 underweight	19	3.86
18.5–24.9 normal weight	305	61.99
25.0–29.9 overweight	131	26.63
>30.0 obese	29	5.89
**MAIN SPORTS**		
Fitness training	197	40.04
Weight training	164	33.33
Bodybuilding	45	9.15
Other sports	83	16.87
**TRAINING YEARS**		
≤ 1	106	21,54
1–2	119	24,19
3–4	157	31,91
≥4	108	21,95
**TRAINING FREQUENCY PER WEEK**		
sporadic	34	6.91
1–2 times	120	24.39
3–4 times	236	47.97
5–6 times	78	15.85
Daily	23	4.67
**TRAINING SESSION DURATION**		
≤ 60 min	145	29.47
60–90 min	238	48.37
≥ 90 min	108	21.95
**NS USERS**	**280**	**56.91**
Male	191	68.21
Female	87	17.68
**HEALTH RELATED RISK ACCEPTANCE OF NS USERS (*****N*** = 276)^*^		
No	154	55.80
Very low	71	25.72
Minor	25	9.06
Partial	16	5.80
Major	7	2.54
Very high	3	1.09

* *280 of all asked fitness studio visitors declared to use NS. 276 of these NS users provided answer to their risk acceptance*.

Of all fitness studio visitors, 53.9% have ≥2 years training experience. About 29.5% of the fitness studio visitors have an average training session ≤ 60 min. The largest group (48.4%) trains between 60 and 90 min, whereas 22.0% trains ≥90 min. The largest group of the participants (*N* = 236) 48.0% trains 3–4 times per week. One Hundred and Twenty athletes (24.4%) stated a frequency of once or twice a week, 78 athletes (15.9%) train 5–6 times a week, followed by the sporadic training group (*N* = 34; 6.9%). Only 23 athletes (4.7%) stated that they train daily. Fitness training was the preferred sports type (40.0%), followed by weight training (33.3%), bodybuilding (9.2%), and other main sports (16.9%), like soccer (3.1%). Missing data were found for eigth athletes (1.6%), therefore it was not possible to calculate BMI and training characteristics and the participants were not considered in the evaluation. Moreover, the athletes were asked about the acceptance of negative health consequences caused by nutrition intake. One Hundred and Twenty Two (44.2%) subjects would accept at least very a low risk.

### Questionnaire

The questionnaire consisted of six major parts. The first two parts asked about general fitness studio and training related characteristics like training frequency, training session duration, training years, as well as competition participation in likert skales. Additionally, the participants were asked to choose between the health orientated training types prevention and rehabilitation training or the performance reason enhancement and preservation as their main sport specific training reason. In the third part, participants were asked about their last month NS-consumption in general. The consumption of specific performance enhancing substances (e.g., creatine, PWB, DMAA) was focused in part four. Consumption prevalence was divided into the last month and all time intake. An adjusted survey of the Federal Institute for Risk Assessment (Germany) (Röder et al., 2013) for peer group specific risk communication of NS, was used to ask for the individual reasons of NS consumption, in part five.

The pre-determined questions are based on qualitative interviews which were held in four unspecific focus groups. The reasons were asked using a Likert scale from 1 (do not agree) to 7 (fully agree) and included 12 pre-determined questions about performance related reasons like, improving physical, mental, and sports performance as well as health related reasons such as compensation for deficiency symptoms of aging, compensation of lifestyle related deficiency symptoms, compensation of natural deficiency symptoms (e.g., iodine deficiency), malnutrition, compensation of weak food quality and improving the quality of life. Additionally, the questionnaire assesses the reasons for NS intake to silence the conscience (e.g., caused by unhealthy eating behavior) and that NS are modern or on-trend (e.g., newly released supplements). Furthermore, we asked for the acceptance of potential health risks caused by NS intake using a Likert scale from 1 (no acceptance) to 6 (very high acceptance). The last part of the questionnaire deals with socio-demographic characteristics including age (5 year steps), gender, educational level and self-reported weight and height. Weight and height was included because PWBs contain both muscle and therefore weight gaining and weight losing ingredients, e.g., creatine, DMAA and Caffeine.

The aim of the study was to determine which of the training specific factors, NS intake reasons, training reasons, as well as social factors and BMI are related to creatine and PWB supplement intake.

### Procedure

The study was approved by the ethics committee of the University of Mainz (Germany). An additional data security approval was obtained by the data protection department of Rhineland-Palatinate (Germany). A self-reported paper and pencil questionnaire was distributed to fitness studio visitors in Mainz (Germany) between May and June 2016. Thirty-six fitness studios were located in Mainz and the surrounding suburbs. All lady fitness studios, electro muscle stimulating training studios, crossfit boxes, and fight club fitness studios were excluded from the study. A total of 26 fitness studios were included and asked personally to participate in the survey. Per request, additional information was sent via email to the fitness studios. Thirteen of the remaining 26 fitness studios with approximately 12,456 members agreed to participate in the study. Four studios are located in the inner city, three in the city center, and six studios in the outer area of Mainz. The data was collected at least for 10 h in every studio. To reach the different types of studio visitors, the survey was implemented for a minimum of 3 h in the morning, afternoon and evening. Overall, 785 fitness studio visitors were asked to fill out the questionnaire. The final sample included surveys from 521 (66.3%) fitness studio members. 29 collected surveys were not included in the data set due to incomplete (or uninterpretable) responses. At the beginning of the questionnaire, athletes were informed about their anonymity and voluntary participation. The participants gave their written consent to participate in the survey. To increase the level of anonymity of the survey, the collection of personal data was reduced to a minimum. Only a German version of the questionnaire was distributed. No sample size calculation was performed because the proportion of individuals consuming PWB was unknown.

### Statistics

An ANOVA was used to analyze the training reasons between NS users and NS non-users. The results were considered statistically significant at *p* ≤ 0.05.

In the second part individual χ^2^-tests were computed to determine group differences between creatine use, PWB use, and PWB use including DMAA, N,α-DEPEA, DMAE, and DMBA with different predictor variables as a pre-analysis to reduce number of variables for the logistic regression analysis.

The variables of training parameters, training reasons, consumption reasons, and acceptance of health related risks were split at their means and recoded in dichotomous ones (e.g., training session duration < 60 min and training session duration > 60 min).

In part three, all remaining significant variables of the previous χ^2^-tests (listed in Table 5) were integrated in logistic regression analysis. Logistic regression analyses were conducted to provide odds ratios (OR) and 95% confidence intervals, for the variables whether they can predict creatine, PWB use, and PWB use containing DMAA or similar agents, to filter risk groups and create prediction patterns. Statistical analyses were calculated with SPSS PASW 23 Statistics (IBM Corp., Somers, NY).

## Results

### Training and NS consumption patterns

The major training reasons of the 492 participants are listed in Table 2. The main reasons by far of NS users for training is performance enhancement (61.4%) which was declared by NS users more often than by non NS users (*p* < 0.001). In contrast, non NS users train more often because of performance preservation (39.2%, *p* = 0.012) and preventive training aims (16.5%, *p* < 0.001) compared to NS users, even though performance enhancement is still among the main reasons for training (37.7%). No differences were found for rehabilitation aspects.

**Table 2:** Main aspect for fitness training of 492 fitness studios visitors.

	**NS users *N* = 280 (%)**	**Non NS users *N* = 212 (%)**	***p*-value**
Prevention	14 (5.0)	35 (16.5)	0.000
Rehabilitation	9 (3.2)	8 (3.8)	0.715
Preservation	81 (28.9)	83 (39.2)	0.012
Performance enhancement	172 (61.4)	80 (37.7)	0.000
Missings	4 (1.4)	6 (2.8)	

Table 3 lists the individual reasons for NS use, as declared by the participants (Likert scale 1–7). Participants who used NS during the last 4 weeks stated the improvement of physical (5.43 ± 1.89) and sports performance (5.29 ± 1.89), as the most important aim for supplementation. Among all NS users, the compensation of malnutrition (4.99 ± 2.16), health beneficial reasons (4.69 ± 1.84), and improved quality of life (4.41 ± 2.02) were important, as well as the intention to increase mental performance (3.75 ± 2.12). Therefore, the performance aspect seems to be the main aim for NS use followed by health related reasons.

**Table 3:** Reasons for NS use (Likert scale 1–7; 1 = do not agree, 7 = fully agree).

**Performance related reasons**	**Mean**	***SD***
Improve physical performance	5.43	1.89
Improve mental performance	3.75	2.12
Improve sports performance	5.29	1.89
**Health related reasons**	**Mean**	***SD***
Health benefits	4.69	1.84
Compensation for deficiency symptoms of aging	2.47	1.88
Compensation of lifestyle related deficiency symptoms (lifestyle)	2.80	1.92
Compensation of natural deficiency symptoms (e.g., iodine deficiency)	3.14	2.13
Malnutrition	4.99	2.16
Groceries do not contain everything you need (food quality)	3.26	2.06
Improve quality of life	4.41	2.02
**Others**	**Mean**	***SD***
Silence the conscience	2.76	2.07
NS are modern and on-trend	1.59	1.28

### General use of NS

**Figure 1:**
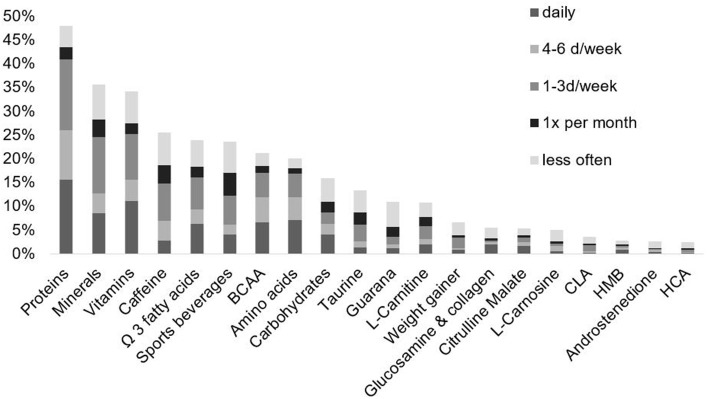
Prevalence and frequency of NS consumption of 492 fitness center visitors.

Among the 492 fitness studio members, 280 (56.9%) of the surveyed participants used NS during the last month. The supplements with the highest use were proteins such as shakes or bars (48.0%), minerals (35.6%), vitamins (34.1%), caffeine (25.6%), Omega-3 fatty acids (24.0%), sports beverages (23.6%), BCAA (21.1%), and amino acids (20.1%). Less than 20% of the fitness studio members use carbohydrate supplements (15.9%), taurine (13.4%), guarana (11.0%), l-carnitine (10.8%), weight gainer (6.7%), glucosamine and collagen (5.5%), citrulline malate (5.3%), l-carnosine (5.1%), CLA (3.7%), HMB (2.8%), androstenedione (2.6%), and HCA (2.4%) (see Figure 1). Detailed consumption data are provided in the Supplemental Table. Male participants are more likely to use NS compared to female participants (*p* < 0.001).

### Creatine and PWB consume

Descriptive statistics were used to show prevalence of creatine and PWB intake. χ^2^-tests and logistic regression analyses were used to asses relations between the sociodemographic and anthropometric characteristics, the training specific parameters and intake reasons with the consumption of creatine, PWB, or PWB containing DMAA or similar agents.

**Figure 2:**
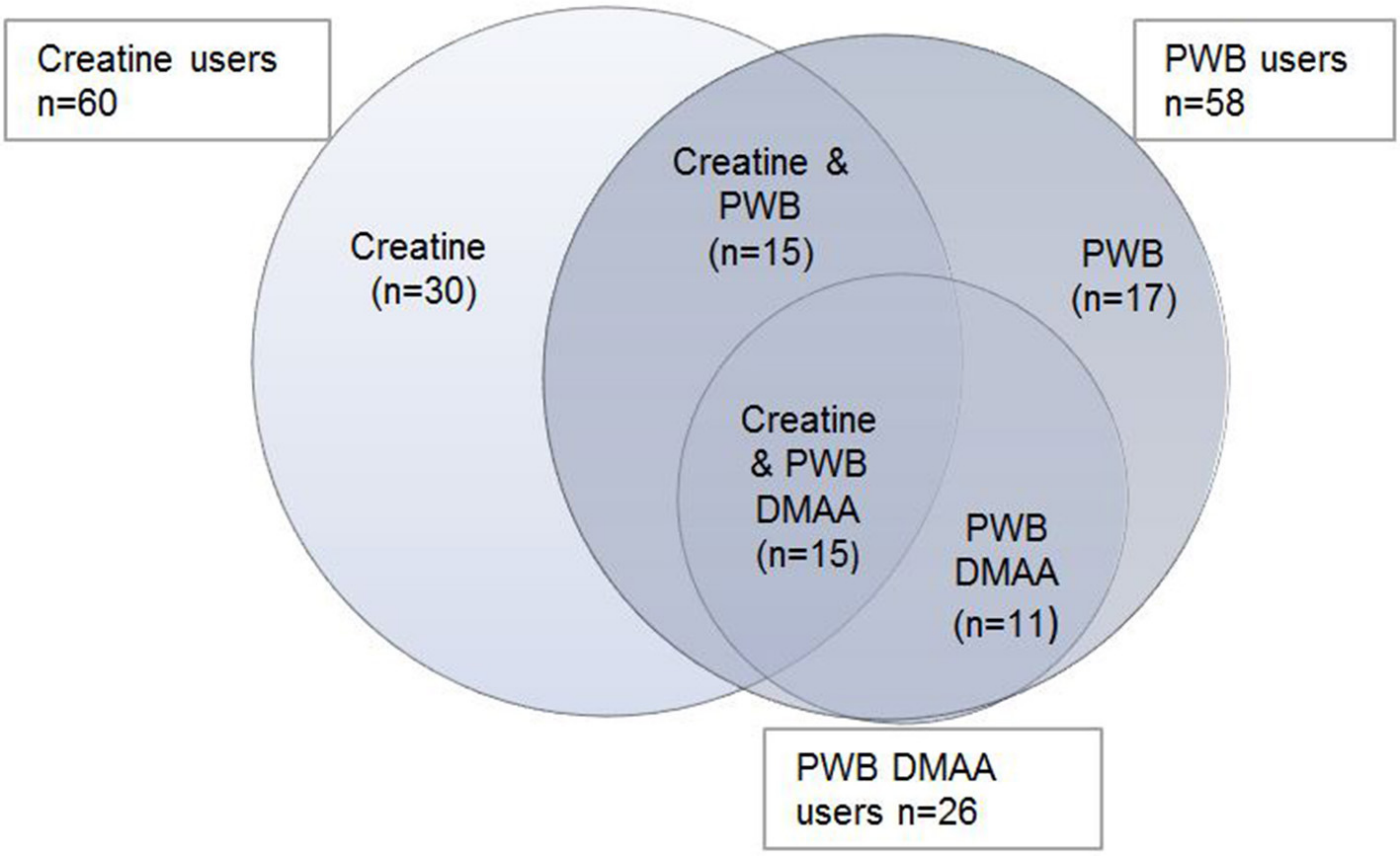
Mixed last month consume of 60 creatine and 58 PWB users.

The all-time prevalence for the use of PWB (45.4% among NS users) was similar to the prevalence of creatine use (50.4%), whereas these NS were consumed during the last month by a total of 21.4% users for creatine and 20.7% users for PWB (see Table 4). When asking for the active search of specific ingredients that must be contained in the PWBs, 38.6% of the PWB users focused on specific substances in PWBs. In our study, different subgroups emerge within the creatine and PWB groups, with overlapping consume during the last month. We found 30 persons who consume creatine only. Fifteen persons reported the intake of creatine and PWB, and 15 stated the use of creatine, PWB, and PWBs with DMAA. On the other hand 17 persons stated a PWB intake and 11 persons just use PWBs with DMAA (see Figure 2). Additionally we found that 43 (33.9%) of the PWB users (*N* = 127) search for caffeine, and 32 (25.2%) subjects for beta alanine. DMAA and similar ingredients were essential for 26 (20.5%) of the PWB users (Table 4). The use of PWBs and creatine was higher among males (*p* < 0.001) (Table 5).

**Table 4:** Prevalence of creatine and pre workout booster (PWB) use.

**Creatine**		***N***	**% of all (*N* = 492)**	**% of NS users (*N* = 280)**	
All time prevalence of consume		141	28.7	50.4	
Consume during the last month		60	12.2	21.4	
**Pre workout-booster**		***N***	**% of all (*****N*** = **492)**	**% of NS users (*****N*** = **280)**	
All time prevalence of consume		127	25.8	45.4	
Consume during the last month		58	11.8	20.7	
		***N***	**% of all (*****N*** = **492)**	**% of NS users (*****N*** = **280)**	**% of PWB users (*****N*** = **127)**
Explicit focus on specific substances		49	10.0	17.5	38.6
	DMAA, N,α-DEPEA, DMAE, DMBA	26	5.3	9.3	20.5
	Synephrine	11	2.2	3.9	8.7
	Niacine	6	1.2	2.1	4.7
	Caffeine	43	8.7	15.4	33.9
	Beta alanine	32	6.5	11.4	25.2
	Other	11	2.2	3.9	8.7

**Table 5:** χ^2^-test to determine group differences of dichotomized basic characteristics and creatine and pre workout booster (PWB) use during the last 4 weeks.

Characteristics^*^	**Creatine**				**Pre workout booster**				**Pre workout booster DMAA**			
	**OR**	**95% CI**	**χ2 (d.f. = 1)**	***p*-value**	**OR**	**95% CI**	**χ2 (d.f. = 1)**	***p*-value**	**OR**	**95% CI**	**χ2 (d.f. = 1)**	***p*-value**
**SOCIAL AND ANTHROPOMETRIC**												
Gender: male	23.494	5.665–97.428	37.7	< 0.001	6.549	2.752–15.586	22.9	< 0.001	17.870	2.401–133.012	14.7	< 0.001
Age > 25	1.658	0.760–3.620		0.227	0.894	0.453–1.766		0.724	0.754	0.292–1.942		0.603
School education: A-level	1.031	0.591–1.797		1.000	1.571	0.901–2.738		0.112	1.445	0.653–3.196		0.408
Job: full time	0.764	0.437–1.334		0.383	1.787	1.023–3.123		0.054	1.377	0.618–3.071		0.527
Student/pupil/apprentice	1.151	0.666–1.990		0.677	1.747	0.987–3.094		0.066	1.121	0.507–2.477		0.842
Family status: single	3.811	1.161–12.509	5.5	0.020	1.570	0.647–3.805		0.429	1.403	0.410–4.797		0.782
Having a child	4.065	1.239–13.331	6.2	0.013	2.823	0.991–8.039		0.054	1.525	0.447–5.208		0.784
BMI ≥ 25	2.655	1.534–4.593	12.8	0.001	1.982	1.123–3.496	5.7	0.022	2.502	1.129–5.546	5.4	0.030
**TRAINING PARAMETERS**												
Training ≥2 years	2.105	1.172–3.780	6.4	0.012	2.239	1.234–4.065	7.3	0.008	2.405	0.992–5.831		0.067
Training frequency ≥2 times a week	32.471	4.455–236.673	28.0	< 0.001	15.146	3.646–62.923	23.8	< 0.001	12.260	1.646–91.325	9.6	0.001
Training session duration ≥ 60 min	1.435	0.762–2.701		0.293	1.230	0.660–2.292		0.646	2.394	0.810–7.073		0.124
Competition participation	0.839	0.419–1.681		0.735	1.392	0.738–2.625		0.303	2.480	1.090–5.643	4.9	0.044
**TRAINING REASONS**												
Strength training and bodybuilding	7.264	3.666–14.391	40.6	< 0.001	2.350	1.333–4.142	9.0	0.004	3.192	1.360–7.493	7.8	0.007
Performance enhancement	4.057	2.090–7.874	19.3	< 0.001	2.949	1.590–5.470	12.6	< 0.001	4.091	1.516–11.036	8.9	0.004
**CONSUMPTION REASONS**												
Health benefit	1.106	0.619–1.978		0.769	1.112	0.617–2.003		0.766	0.601	0.260–1.388		0.295
Lifestyle	1.160	0.650–2.069		0.659	1.513	0.840–2.723		0.180	1.664	0.734–3.771		0.301
Quality of life	3.744	1.939–7.232	16.7	< 0.001	1.885	1.027–3.459		0.052	1.486	0.648–3.404		0.411
Natural deficiency symptoms	1.204	0.669–2.168		0.553	0.963	0.529–1.751		1.000	1.953	0.837–4.557		0.148
Malnutrition	1.617	0.903–2.893		0.110	1.052	0.589–1.878		0.883	0.968	0.432–2.172		1.000
Food quality	1.432	0.800–2.561		0.244	1.592	0.879–2.881		0.140	2.127	0.892–5.072		0.100
Aging deficiency symptoms	1.091	0.615–1.936		0.773	1.149	0.640–2.063		0.658	1.562	0.682–3.576		0.310
Sports performance	9.084	3.375–22.006	31.2	< 0.001	10.400	4.001–27.035	31.6	< 0.001	1.844	1.645–2.067	20.3	< 0.001
Physical performance	6.877	3.579–13.212	38.8	< 0.001	5.713	3.002–10.872	31.8	< 0.001	2.196	0.968–4.982		0.061
Mental performance	3.357	1.853–6.083	16.8	< 0.001	3.576	1.945–6.574	17.9	< 0.001	3.353	1.435–7.835	8.5	0.005
Silence the conscience	2.188	1.176–4.070	6.2	0.016	2.338	1.245–4.393	7.1	0.010	2.539	1.029–6.263	4.3	0.040
Modern and on-trend	1.278	0.670–2.437		0.498	1.358	0.710–2.599		0.391	1.370	0.567–3.311		0.481
**ACCEPTANCE OF HEALTH RELATED RISKS**												
Risk-taking	2.612	1.442–4.730	10.4	0.002	2.318	1.272–4.225	7.7	0.007	3.874	1.571–9.551	9.7	0.003

* *The χ^2^-values of all relationships were significant at a level of p ≤ 0.05*.

#### Chi square

χ^2^-tests were performed to study group differences between the dependent variable creatine use (*N* = 60), PWB use (*N* = 58) and PWB use containing DMAA (*N* = 26) or similar agents, in relation to the dichotomized independent variables summarized in Table 5. The use of creatine was higher among males compared to females. Other social variables like having a child and not being married also showed significance. Focusing on anthropometrics, only a BMI ≥25 was related to creatine intake, which might be an indicator for higher muscle mass in this specific population. Furthermore, creatine intake is related to a higher training experience and more frequent training. Similarly, strength training and bodybuilding as practiced disciplines, as well as performance enhancement training reasons are further indicators for creatine consumption. Health related reasons for NS intake, like increasing quality of life or silencing the conscience show significant group differences. Athletes who specify sports related, physical, and mental performance aspects are related to higher creatine intake. The acceptance of negative health consequences is hazarded more likely by creatine users.

The consumption of PWB was significantly related to gender (male) and a higher BMI, more than 2 training years and more than 2 training sessions per week. Similar to creatine intake, strength training and bodybuilding, as well as performance enhancement as reasons for training are accompanied with PWBs use. All performance increasing aspects as reasons for NS use (physical performance, sports performance, and mental performance) seem to predict a higher possibility for PWB intake. Additionally, the NS consumption reason to silence the conscience, and an acceptance of health-related risks are predictors for PWB intake.

The subgroup of PWBs containing DMAA and similar ingredients are more often used by men and overweight athletes. The type of supplement is more frequently used by subjects who train more than twice a week and take part in competitions. Similar to both other supplements the use of PWBs containing DMAA or similar agents was higher in the group of participants who refer strength training and bodybuilding, as well as performance enhancement training reasons as the reasons for training. Furthermore, fitness studio visitors who want to increase their sports or mental performance by NS use more often use PWB products containing these pharmacologically active substances. Finally, the group of fitness studio visitors who stated a higher focus to silence the conscience as a reason for NS use, as well as the group of participants who stated a higher health risk acceptance were more likely to use PWBs including DMAA or similar agents.

#### Logistic regression analysis

A stepwise logistic regression was performed for each of the dependent variables creatine use, PWB use, and PWB use including DMAA or similar agents, to determine potential significant interaction effects of previously found significant variables. Variables with a *p*-value ≤ 0.05 were considered to be statistically significant. Estimated odds ratio (OR) with the 95% confidence interval (CI) for each supplement and their predictors are shown in Table 6.

**Table 6:** ORs with 95% confidence intervals for each significant predictor variable.

Characteristics*	**Creatine**			**Pre workout booster**			**Pre workout booster DMAA**		
	**OR**	**95% CI**	***p*-value**	**OR**	**95% CI**	***p*-value**	**OR**	**95% CI**	***p*-value**
Gender: male	13.516	2.589–70.572	0.002	2.837	0.998–8.062	0.050	9.002	1.006–80.583	0.049
Training frequency ≥2 times a week				9.253	1.126–76.023	0.038			
Competition participation							4.369	1.352–14.119	0.014
Strength training and bodybuilding	4.478	1.710–11.727	0.002						
Quality of life	2.822	1.050–7.587	0.040						
Sports performance	3.872	1.009–14.851	0.048	6.290	1.659–23.846	0.007			
Physical performance	2.969	1.047–8.414	0.041						
Mental performance							3.466	1.157–10.387	0.026

Dependent variables are creatine and pre workout booster (PWB) use during the last 4 weeks. ^*^Predictors were significant at a level of p ≤ 0.05.

Regarding creatine, for male participants the odds is 13.5-fold larger than for female exercisers (CI: 2.589–70.572). A positive relation for creatine intake was confirmed for the training types, strength training and bodybuilding (OR: 4.5; CI: 1.710–11.727). Exercisers who want to increase their life quality (OR: 2.8; CI: 1.050–7.587), sports performance (OR: 3.9; CI: 1.009–14.851) and physical performance (OR: 3.0; CI: 1.047–8.414) have a slightly increased OR to take creatine. The current model coefficient testing vs. the baseline model shows significance χ^2^ = 100.6, d.f. = 14, *p* < 0.001.

In the second regression, fitness studio visitors who stated a training frequency of more than twice a week showed a 9.3-fold greater chance to use PWBs (CI: 1.126–76.023). The relation was estimated with an OR of 6.3 between the intake of PWBs and the aim to increase sports performance (CI: 1.659–23.846). Just a very weak relation was found for gender (males) (OR: 2.8; CI 0.998–8.062). Omnibus test of the current model was significant (χ^2^ = 68.4, d.f. = 11, *p* < 0.001).

Within the third regression analysis, a significant relationship between PWBs containing DMAA or similar agents was found. For male gender, competition participation and increase of mental performance capacity showed OR: 9.0 (CI: 1.006–80.583), OR: 4.4 (CI: 1.352–14.119) and OR: 3.5 (CI: 1.157–10.387), respectively. The test of the current model against the baseline model shows significance as well χ^2^ = 54.4, d.f. = 10, *p* < 0.001.

## Discussion

The aim of the study was to determine the prevalence of NS use, training characteristics and the intake reasons among fitness studio visitors in Mainz with a special emphasis on new multi-ingredient based PWBs, of which some contain stimulants such as DMAA, N,α-DEPEA, DMAE, and DMBA.

The most consumed NS in fitness studios were protein supplements such as shakes or bars (see Figure 1), used more or less regularly by approximately half of the fitness studio visitors, followed by minerals and vitamins. These results are comparable to data reported in the literature with a wide range between 28 and 50.0% for protein supplements and between 11.8 and 45.8% for minerals and vitamins (Morrison et al., 2004; Oliver et al., 2008; Goston and Correia, 2010; Bianco et al., 2011; El Khoury and Antoine-Jonville, 2012; Hildebrandt et al., 2012; Saeedi et al., 2013). In contrast, we found a higher prevalence for caffeine containing supplements such as energy drinks or caffeine tablets with 25.6% compared to 8.6% (El Khoury and Antoine-Jonville, 2012). This difference might be influenced by the intake of PWBs which contain caffeine and were not common in older studies (Eudy et al., 2013).

The use of the performance enhancing supplement creatine, which is often an ingredient in PWBs as well, shows intake prevalence in fitness studio visitors between 8.0 (Goston and Correia, 2010) and 48.3% (Bianco et al., 2011). With an all-time prevalence of fitness studio visitors for creatine use of 28.7%, and prevalence of 12.2% for the last 4 weeks, our results are in between this range.

We found five predictors for creatine use which are gender, sports type strength training and bodybuilding, the NS intake reasons increasing physical and sports performance, as well as increasing quality of life. The positive relation of creatine use with gender is in accordance with Froiland et al. (2004), Saeedi et al. (2013), and Fraczek et al. (2016), who described significantly higher intake in males than in females and differences between various sports. The relation with gender is supposedly directly linked to the other strongly related factors, which we found to be related to the male gender as well. This can be explained by the effect that creatine intake increases muscle strength, muscle mass, energy, and performance, which reportedly are no primary training reason for most women, who usually have a higher health related focus (Cooper et al., 2012; Saeedi et al., 2013). However, these effects outline the main training reasons for bodybuilders and strength athletes, who often use creatine to increase muscles strength, lean body mass and to enhance athletic performance (Vandenberghe et al., 1997; Feldman, 1999; Morrison et al., 2004). Furthermore, we found a positive relation between creatine intake and the aim to increase the quality of life and well-being. These effects are described in the literature for elderly people who try to avoid a decrease in functional performance in everyday tasks (Moon et al., 2013). The aspect of performance enhancement in combination with the general well-being through physical activity which was shown by Penedo and Dahn (2005) might be responsible for this relation. In contrast, no relation was found for PWBs and well-being respectively quality of life. This might be explained by Spradley et al. (2012) who described improved perceived feeling of energy, focus and alertness after stimulant intake. But a rapid subsidence of the stimulating effects which causes a so called “booster crash” and effects well-being negatively after training.

For PWB intake we found three predictors, which are gender (male), training frequency ≥2 times a week and sports performance. For PWBs including DMAA or similar agents the variables gender (male) competition participation and the increase of mental performance showed significance. The prevalence for creatine and PWB use were comparable for both, the all-time and the last month consumption. Similarly to creatine consumption, the gender (male) was related to the use of PWBs and PWBs containing DMAA or similar substances. Again, this is probably, because among fitness studio visitors, the training reason performance enhancement is mainly described for males (Froiland et al., 2004; Saeedi et al., 2013; Fraczek et al., 2016). Additionally, PWBs often contain creatine which is a performance enhancing ingredient and has effects on muscle growth (Eudy et al., 2013). One predictor for the use of PWB, but not for creatine was a high training frequency, over two times a week, which has been shown for general NS use in fitness studios (Oliver et al., 2008; Goston and Correia, 2010; El Khoury and Antoine-Jonville, 2012). The use of PWBs is proposed to reduce fatigue caused by prior training sessions and to facilitate mental and cognitive performance during the training (Sato et al., 2012; Spradley et al., 2012). This effect has been reported for the intake of large doses of energy drinks sharing some of the ingredients, like caffeine, with PWB (Richards and Smith, 2016). Because of the large dose of cognitive and mental supporting ingredients, it is supposedly easier for the fitness studio visitor to focus on training (Spradley et al., 2012). Furthermore, the possible ingredients synephrine, and picamilon, which were developed to provide anti-anxiety and anti-convulsive effects reviewed by Avula et al. (2016) could be used to additionally explain the intake aim to enhance mental performance, with an elevation of mood (Richards and Smith, 2016). In a previous study, students studying in sports-related fields show highest supplement intake for cognitive enhancing products (Dietz et al., 2013). Additionally, the intake might be caused by social interactions with peers or other fitness studios visitors who recommend the PWBs and non-experienced exercisers may be prone to the advice of the stronger and established gym members. However, no relation was found for the predictor variable training years in the logistic regression. In contrast to creatine, we found a relation between the use of PWBs containing DMAA or similar substances and the factor competition participation and mental performance. Among elite athletes who compete under the anti-doping code of the World Anti-Doping Agency (WADA), the use of DMAA was not uncommon (Institut für Biochemie, 2017) and might still be used with the intention to increase sports and mental performance. Despite the ban of the substance by the WADA in 2010, the number of positive DMAA related doping test results increased from 123 in 2010 to 320 adverse analytical findings in 2012, which were 45% of all positive test results in the group prohibited stimulants (Institut für Biochemie, 2017).

As shown in this study, fitness center visitors often use different kinds of NS simultaneously, even though the long term effect of mixed consume of different NS is not yet investigated. Additionally, larger studies including women are required and further toxicological markers and cardiac function must be critically evaluated to gain a more comprehensive insight into the physiological effects of PWBs. The risk profile of many substances included in PWBs and especially their combinations have not yet been evaluated in detail and were not tested sufficiently in long term (Kim et al., 2011; Cohen et al., 2016; Richards and Smith, 2016; Dunn, 2017). In a study from 2013, controlled use of DMAA did not show measurable adverse effects (Schilling et al., 2013).

However, it cannot be expected that the results of DMAA are identical to comparable substances such as BMPEA or DMBA, of which the safety profile has never been studied in humans, especially in combination with other substances (Cohen et al., 2015, 2016). The fact that substances without a safety profile are freely purchasable as nutritional supplements is at least worrying. Furthermore, only few PWBs list the single ingredients and their dosages, whereas most PWBs label their ingredients as a “proprietary blend” (Eudy et al., 2013) and do not indicate their dosages. Especially information on interactions of the different substances in combined products is scant, inconclusive, or conflicting (Eudy et al., 2013). Therefore, not clearly declaring ingredients in supplements is an additional risk factor.

Tian et al. (2009) described an unawareness of 86.4% of university athletes that supplements can adversely affect health, and only 29.5% were confident not to contravene sports doping regulations. This described lack of information regarding to active ingredients can increase probability for adverse intake effects and unintentional overdosing (Eudy et al., 2013). Surprisingly, about 80% of the NS consumers would take no or just a very low risk through NS intake and potential risk taking did not remain in the final logistic regression, which is controversial to the potential harms caused by PWBs.

The study results are limited to the specific population group of fitness study visitors. In different population groups like competitive athletes, recreational athletes from different sports, or non-athletes the prevalence might be different. Another potential restriction is caused by the use of a self-administered questionnaire for sensitive questions for physiologically active substances. Therefore, true prevalence might be higher. It should be considered that logistic regression analysis cannot prove final causal relations between the dependent and the independent variable in the used model. Additionally, because of the restricted number of surveyed questions, the analysis does not cover all possible reasons and aspects. In future studies open response questions should be added to enable further relevant aspects for training and intake reasons. Because the knowledge about prevalence and intake reasons of PWBs are limited for sociodemographic and anthropometric characteristics, training characteristics, and reasons for NS use and fitness training we obtained a widespread but more unspecific first impression. For example asking for motivational reasons like “physical appearance” would have helped to cover other aspects reliably. Further studies should focus on training and intake motives for a better understanding of motivational aspects with standardized instruments.

## Conclusion

Despite NS may serve as a practical assistance to meet sports specific goals, evidence shows that NS are typically not required if athletes have a balanced diet. Exercisers in fitness studios consume a large number of different supplements like creatine and PWB, some of which contain stimulants such as DMAA, N,α-DEPEA, DMAE, and DMBA to increase sports performance capacity. The current findings show surprisingly high intake prevalence for PWB supplements. Reasons for consumption seem to be slightly different from creatine use, especially if the users are searching for stimulating ingredients. The results might help to create a consumption pattern and support to identify potential endangered fitness studio visitors for prevention and risk communication of NS and especially PWBs. Especially the mixed consumption of NS might have adverse effects and hides potential risks and harms. Therefore, the study of long-term effects and the enlightenment of fitness studio visitors about the cost-benefit ratio is of unique importance.

## Author contributions

MD, PS, TE, and EN designed the experiment. MD and EN analyzed the study results. PS and TE helped in data analysis. MD and EN wrote the article and all authors interpreted and discussed the results.

### Conflict of interest statement

The authors declare that the research was conducted in the absence of any commercial or financial relationships that could be construed as a potential conflict of interest.
