# Supplementary material for: Boost Me: Prevalence and Reasons for the Use of Stimulant Containing Pre Workout Supplements Among Fitness Studio Visitors in Mainz (Germany)
Source: Front Psychol. 2018 Jul 17;9:1134. doi: 10.3389/fpsyg.2018.01134 (PMC6056667; doi:10.3389/fpsyg.2018.01134)
Supplement: Supplementary file 1 [file Table_1.DOCX]

Supplemental table: Prevalence and frequency of NS consumption a) total numbers, b) percentages

a)

| (%) | No. | Supplement | daily | 4-6 d/week | 1-3d/week | 1x per month | less often | never | user total | total |
| --- | --- | --- | --- | --- | --- | --- | --- | --- | --- | --- |
| 48.0 | 236 | Proteins | 77 | 51 | 73 | 13 | 22 | 256 | 236 | 492 |
| 35.6 | 175 | Minerals | 42 | 21 | 58 | 18 | 36 | 317 | 175 | 492 |
| 34.1 | 168 | Vitamins | 55 | 22 | 47 | 11 | 33 | 324 | 168 | 492 |
| 25.6 | 126 | Caffeine | 14 | 20 | 39 | 19 | 34 | 366 | 126 | 492 |
| 24.0 | 118 | Ω 3 fatty acids | 31 | 15 | 33 | 11 | 28 | 374 | 118 | 492 |
| 23.6 | 116 | Sports beverages | 20 | 10 | 30 | 24 | 32 | 376 | 116 | 492 |
| 21.1 | 104 | BCAA | 33 | 26 | 25 | 7 | 13 | 388 | 104 | 492 |
| 20.1 | 99 | Amino acids | 35 | 24 | 24 | 6 | 10 | 393 | 99 | 492 |
| 15.9 | 78 | Carbohydrates | 20 | 11 | 12 | 11 | 24 | 414 | 78 | 492 |
| 13.4 | 66 | Taurine | 7 | 6 | 17 | 13 | 23 | 426 | 66 | 492 |
| 11.0 | 54 | Guarana | 6 | 4 | 8 | 10 | 26 | 438 | 54 | 492 |
| 10.8 | 53 | L-Carnitine | 10 | 5 | 14 | 9 | 15 | 439 | 53 | 492 |
| 6.7 | 33 | Weight gainer | 4 | 2 | 11 | 2 | 14 | 459 | 33 | 492 |
| 5.5 | 27 | Glucosamine & collagen | 10 | 2 | 2 | 2 | 11 | 465 | 27 | 492 |
| 5.3 | 26 | Citrulline Malate | 8 | 4 | 5 | 2 | 7 | 466 | 26 | 492 |
| 5.1 | 25 | L-Carnosine | 3 | 5 | 3 | 2 | 12 | 467 | 25 | 492 |
| 3.7 | 18 | CLA | 1 | 2 | 6 | 2 | 7 | 474 | 18 | 492 |
| 2.8 | 14 | HMB | 4 | 3 | 1 | 2 | 4 | 478 | 14 | 492 |
| 2.6 | 13 | Androstenedione | 2 | 2 | 1 | 1 | 7 | 479 | 13 | 492 |
| 2.4 | 12 | HCA | 1 | 1 | 2 | 2 | 6 | 480 | 12 | 492 |
|  |  |  |  |  |  |  |  |  |  |  |
| b) |  |  |  |  |  |  |  |  |  |  |
|  |  |  | % | % | % | % | % | % | % | % |
| (%) | No. |  | daily | 4-6 d/week | 1-3d/week | 1x per month | less often | never | user total | total |
| 48.0 | 236 | Proteins | 15.65 | 10.37 | 14.84 | 2.64 | 4.47 | 52.03 | 47.97 | 100 |
| 35.6 | 175 | Minerals | 8.54 | 4.27 | 11.79 | 3.66 | 7.32 | 64.43 | 35.57 | 100 |
| 34.1 | 168 | Vitamins | 11.18 | 4.47 | 9.55 | 2.24 | 6.71 | 65.85 | 34.15 | 100 |
| 25.6 | 126 | Caffeine | 2.85 | 4.07 | 7.93 | 3.86 | 6.91 | 74.39 | 25.61 | 100 |
| 24.0 | 118 | Ω 3 fatty acids | 6.30 | 3.05 | 6.71 | 2.24 | 5.69 | 76.02 | 23.98 | 100 |
| 23.6 | 116 | Sports beverages | 4.07 | 2.03 | 6.10 | 4.88 | 6.50 | 76.42 | 23.58 | 100 |
| 21.1 | 104 | BCAA | 6.71 | 5.28 | 5.08 | 1.42 | 2.64 | 78.86 | 21.14 | 100 |
| 20.1 | 99 | Amino acid | 7.11 | 4.88 | 4.88 | 1.22 | 2.03 | 79.88 | 20.12 | 100 |
| 15.9 | 78 | Carbohydrate | 4.07 | 2.24 | 2.44 | 2.24 | 4.88 | 84.15 | 15.85 | 100 |
| 13.4 | 66 | Taurine | 1.42 | 1.22 | 3.46 | 2.64 | 4.67 | 86.59 | 13.41 | 100 |
| 11.0 | 54 | Guarana | 1.22 | 0.81 | 1.63 | 2.03 | 5.28 | 89.02 | 10.98 | 100 |
| 10.8 | 53 | L-Carnitine | 2.03 | 1.02 | 2.85 | 1.83 | 3.05 | 89.23 | 10.77 | 100 |
| 6.7 | 33 | Weight Gainer | 0.81 | 0.41 | 2.24 | 0.41 | 2.85 | 93.29 | 6.71 | 100 |
| 5.5 | 27 | Glucosamine & collagen | 2.03 | 0.41 | 0.41 | 0.41 | 2.24 | 94.51 | 5.49 | 100 |
| 5.3 | 26 | Citrulline Malate | 1.63 | 0.81 | 1.02 | 0.41 | 1.42 | 94.72 | 5.28 | 100 |
| 5.1 | 25 | L-Carnosine | 0.61 | 1.02 | 0.61 | 0.41 | 2.44 | 94.92 | 5.08 | 100 |
| 3.7 | 18 | CLA | 0.20 | 0.41 | 1.22 | 0.41 | 1.42 | 96.34 | 3.66 | 100 |
| 2.8 | 14 | HMB | 0.81 | 0.61 | 0.20 | 0.41 | 0.81 | 97.15 | 2.85 | 100 |
| 2.6 | 13 | Androstenedione | 0.41 | 0.41 | 0.20 | 0.20 | 1.42 | 97.36 | 2.64 | 100 |
| 2.4 | 12 | HCA | 0.20 | 0.20 | 0.41 | 0.41 | 1.22 | 97.56 | 2.44 | 100 |
